# Reply to Dziewas, R.; Bath, P.M. Endpoints in Dysphagia Trials. Comment on “Speyer et al. Neurostimulation in People with Oropharyngeal Dysphagia: A Systematic Review and Meta-Analyses of Randomised Controlled Trials—Part I: Pharyngeal and Neuromuscular Electrical Stimulation. *J. Clin. Med.* 2022, *11*, 776”

**DOI:** 10.3390/jcm11123403

**Published:** 2022-06-14

**Authors:** Renée Speyer, Anna-Liisa Sutt, Liza Bergström, Shaheen Hamdy, Bas Joris Heijnen, Lianne Remijn, Sarah Wilkes-Gillan, Reinie Cordier

**Affiliations:** 1 Department Special Needs Education, Faculty of Educational Sciences, University of Oslo, 0318 Oslo, Norway; 2 Curtin School of Allied Health, Faculty of Health Sciences, Curtin University, Perth, WA 6102, Australia; reinie.cordier@northumbria.ac.uk; 3 Department of Otorhinolaryngology and Head and Neck Surgery, Leiden University Medical Centre, 1233 ZA Leiden, The Netherlands; b.j.heijnen@lumc.nl; 4 Critical Care Research Group, The Prince Charles Hospital, Brisbane, QLD 4032, Australia; annaliisasp@gmail.com; 5 School of Medicine, University of Queensland, Brisbane, QLD 4072, Australia; 6 Remeo Stockholm, 128 64 Stockholm, Sweden; liza.bergstrom@regionstockholm.se; 7 Speech Therapy Clinic, Danderyd University Hospital, 182 88 Stockholm, Sweden; 8 GI Sciences, School of Medical Sciences, Faculty of Biology, Medicine and Health, University of Manchester, Manchester M13 9PL, UK; shaheen.hamdy@manchester.ac.uk; 9 School of Allied Health, HAN University of Applied Sciences, 6525 EN Nijmegen, The Netherlands; lianne.remijn@han.nl; 10 Discipline of Occupational Therapy, Sydney School of Health Sciences, Faculty of Medicine and Health, The University of Sydney, Sydney, NSW 2006, Australia; sarah.wilkes-gillan@sydney.edu.au; 11 Department of Social Work, Education and Community Wellbeing, Faculty of Health & Life Sciences, Northumbria University, Newcastle upon Tyne NE7 7XA, UK

Our systematic review and meta-analysis of pharyngeal electrical stimulation (PES) and neuromuscular electrical stimulation (NMES) in patients with oropharyngeal dysphagia (OD) is the first paper (Part I) [1] of two companion papers. The second paper (Part II) [2] reports on brain stimulation (i.e., rTMS and tDCS). In addition, a third paper [3] has been published, reporting on behavioural interventions in people with OD. All three systematic reviews use similar methodologies and are based on the Preferred Reporting Items for Systematic Reviews and Meta-Analyses (PRISMA) 2020 statement and checklist [4,5]. The overall aim of the three reviews is to determine the effects of non-invasive brain stimulation (NIBS) techniques and behavioural interventions for people with OD and to provide an overview of the findings based on the highest level of evidence, namely conducting meta-analyses of randomised controlled trials (RCTs).

In a commentary about our review on PES and NMES (Part I), Dziewas and Bath argue that our review did not provide the full picture regarding the treatment of PES [6]. The authors list four arguments, which this current paper will address consecutively:

1. First, Dziewas and Bath state that we excluded two of eight identified randomised-controlled trials of PES (Dziewas et al. [2018] [7] and Suntrup et al. [2015] [8]) because we assumed that there was ‘no confirmation of OD diagnosis prior to treatment’ in these studies. We would like to clarify that we did not exclude these two studies from our systematic review. Both studies met all eligibility criteria; however, they were excluded from the meta-analysis based on the need to reduce heterogeneity of the measures used to report on outcomes between studies when conducting meta-analyses. As such, we report on both studies in detail in our outcome tables (Tables 2 and 3), including their use of fibreoptic evaluation of swallowing recordings (FEES) as part of their protocol.

As summarised in the results section, of the ten studies that met the eligibility criteria of this review, eight studies compared PES to a sham version of the treatment, while two studies compared PES with other types of neurostimulation. Of these ten studies, five studies were excluded from the meta-analyses. We excluded these studies from meta-analyses due to an overlap in the participant population between studies, insufficient data reported for meta-analyses, a lack of confirmation of OD diagnosis by instrumental assessment prior to treatment and to ensure that the outcome data are as homogenous as possible. The studies by Dziewas et al. (2018) [7] and Suntrup et al. (2015) [8] were excluded from meta-analyses for reasons of heterogeneity, as clearly described in the discussion:

*For instance, meta-analyses based on both patients’ self-reported health-related quality of life and visuoperceptual evaluation of instrumental assessments would very likely lead to inappropriate estimated overall effects. Thus, to reduce heterogeneity between outcome measures, some studies were excluded from the meta-analysis. This strong focus on reducing heterogeneity between studies when performing meta-analysis also implies that data other than the authors’ primary outcomes may have been preferably included in this analysis. For example, the primary outcome for Dziewas, Stellato, Van Der Tweel, Walther, Werner, Braun, Citerio, Jandl, Friedrichs, Nötzel, Vosko, Mistry, Hamdy, McGowan, Warnecke, Zwittag and Bath [59] and Suntrup, Marian, Schröder, Suttrup, Muhle, Oelenberg, Hamacher, Minnerup, Warnecke and Dziewas [64] was readiness for decannulation, which was considered too different from outcomes in the other included studies.*

As stated under the Methods section, when selecting outcome measures for inclusion in the meta-analysis, reducing heterogeneity between studies was a priority. If heterogeneity is not within reasonable limits, the results of meta-analyses cannot be adequately interpreted [9]. Therefore, in line with current guidelines on conducting meta-analyses, outcomes that are too disparate should not be combined in meta-analyses as unequivocal differences in effects may be obscured [9]. 

On the continuum of the heterogeneity of dysphagia treatment outcomes, we prioritised visuoperceptual measures of instrumental assessment (VFSS and FEES). If no visuoperceptual measure was available, we considered non-instrumental clinical assessments (e.g., oral intake measures). Oral intake measures were only included if no other clinical data were available, whereas screening tools and patient self-report measures were excluded from the meta-analyses altogether. We considered ‘decannulation’ vastly different from any of the other outcome parameters, and, therefore, based on the guidelines for conducting meta-analyses, decided not to include both trachea studies [7,8].

The core issue at hand is the argument put forward by Dziewas and Bath to group patients with dysphagia who have been tracheotomised using readiness for decannulation as an outcome with patients who have not been tracheotomised using visual perceptual measures of FEES or videofluoroscopy (VFSS) to evaluate dysphagia severity in the same meta-analysis. To us, and we suspect for many others in the dysphagia field, the outcomes used are clearly very different and should therefore not be combined in the same meta-analysis under the principle of avoiding heterogeneity when conducting a meta-analysis to determine the effects of PES.

2. Second, Dziewas and Bath state in their commentary that we focussed on the effects of PES using the Penetration Aspiration Scale (PAS) and ignored relevant clinical measures, such as the Dysphagia Severity Rating Scale (DSRS). VFSS and FEES are widely supported in the literature as ‘gold standard’ assessments in the diagnosis of OD and determining OD severity. This view was confirmed in the recently published whitepaper by the European Society for Swallowing Disorders on screening and non-instrumental assessment for dysphagia [10]. This supported our argument for using PAS data (a visuoperceptual measure of FEES and VFSS) over DSRS data (an oral intake measure [11,12]) as a preferred outcome measure when conducting meta-analysis. Therefore, the decision to only include PAS data for meta-analysis was made for two reasons: (i) all five PES studies eligible for meta-analyses used PAS as an outcome measure, thus ensuring the included outcome measures were homogenous, and (ii) PAS, as opposed to the DSRS, is a visuoperceptual measure of ‘gold standard’ assessments.

Despite our argument for only including PAS outcome data, to be fastidious, we conducted the PES within- and between-group meta-analysis again to determine the differences between the results when including non-instrumental clinical assessment data (i.e., DSRS) with PAS data, against using PAS data only. Two [Jayasekeran et al. (2010) and Vasant et al. (2016)] of the original five studies included in the meta-analyses provided both DSRS and PAS data. 

When conducting meta-analyses based on combined PAS and DSRS data, the following results were found for a *between-group comparison*: using a random-effects model, an overall non-significant no effect between-group total effect size favouring PES was found (*z*(4) = 0.825, *p* = 0.410, Hedges’ *g* = 0.109, and 95% CI = −0.150–0.367). In comparison, the original results, as reported in Speyer et al. (2022), were a non-significant no effect between-group total effect size favouring PES (*z*(4) = 0.718, *p* = 0.473, Hedges’ *g* = 0.099, and 95% CI = −0.170–0.368).

When conducting meta-analyses based on combined PAS and DSRS data, the following results were found for a *within-group comparison*: using a random-effects model, an overall significant moderate within-group total effect size favouring post-intervention was found (*z*(4) = 4.562, *p* < 0.001, Hedges’ *g* = 0.591, and 95% CI = 0.337–0.844). In comparison, the original results, as reported in Speyer et al. (2022), were an overall significant moderate within-group total effect size favouring post-intervention (*z*(4) = 3.983, *p* < 0.001, Hedges’ *g* = 0.527, and 95% CI = 0.268–0.786). In summary, for both within-group and between-group analysis, even if we had included DSRS data instead of PAS data for two of the five studies, the substantive results would have been the same as those reported in our meta-analysis.

3. Third, in their commentary, Dziewas and Bath dispute our conclusions in relation to the interpretation of our reported effects for PES. When conducting meta-analyses, we found significant, large pre-post treatment effects for NMES and moderate pre-post treatment effects for PES. In line with our results, we suggested:

*‘… that NMES may have more promising effects compared to PES. However, NMES studies showed high heterogeneity in protocols and experimental variables, the presence of potential moderators, and inconsistent reporting of methodology. Therefore, only conservative generalisations and interpretation of meta-analyses could be made’.*

We formulated our conclusions with great caution. We disagree with the statement by Dziewas and Bath that interventions can only be compared within the same meta-analysis. When direct comparisons are unavailable, an indirect comparison meta-analysis should evaluate the magnitude of treatment effects across studies [13]. As such, the presentation of the effect sizes of PES and NMES presents a legitimate first step in comparing intervention efficacy.

However, to be prudent, we conducted an additional between-subgroup meta-analysis including all NMES and PES studies in the same meta-analysis to compare the effect sizes between both types of neurostimulation. Between-subgroup analyses using a random-effects model showed significant differences between NMES and PES (*p* = 0.011) with a small effect size in favour of NMES (Hedges’*g* = 0.269; 95% CI = 0.061–0.477; *z*-value = 2.537). These results support our previous cautious conclusions formulated in our review in favour of NMES.

4. Fourth, Dziewas and Bath also argue that interventions need to be cost-effective and that this information is missing from our meta-analysis. We are somewhat surprised by this argument as the purpose of our systematic reviews was clearly not to target cost-effectiveness. We agree that the cost-effectiveness of interventions is important, but this topic is outside the scope of our current publication on treatment effects. Combining clinical dysphagia outcomes with measures of cost-effectiveness in our meta-analyses would have introduced unacceptable heterogeneity.

In summary, we disagree with the arguments put forward by Dziewas and Bath. Reducing heterogeneity in meta-analyses is essential. By ignoring heterogeneity, the results from meta-analyses are not trustworthy and do not contribute to the research or, in this case, evidence-based dysphagia care. We provided an objective overview of the highest level of evidence (meta-analyses of RCTs) in the treatment of dysphagia. Our conservative conclusions are based on meta-analyses taking into account the heterogeneity between studies. 

The final statement by Dziewas and Bath that it is very likely that both NMES and PES are effective and have a clinical role may be true; however, this needs to be confirmed by further research using robust methodologies, as suggested in our reviews. In particular, discussion around outcome measurement of dysphagia severity needs further consideration and ongoing debate. Further, to facilitate comparisons of studies and determine intervention effects, there is a need for more randomised controlled trials with larger sample sizes and greater standardisation of protocols and guidelines for reporting. Finally, while we appreciate the authors’ candour in reporting that one of the authors received financial compensation for being a board member of Phagenesis Ltd., a manufacturer of PES technology, it is worth pointing out that there is a need for further research involving independent researchers without any conflict of interest to replicate the current evidence of PES as an important intervention in tackling the challenges associated with dysphagia. 
